# Prevalence and factors related to psychological distress among ethnic minority adults in a semi-modern village in rural Vietnam: an evolutionary mismatch framework

**DOI:** 10.1093/emph/eoab014

**Published:** 2021-04-30

**Authors:** Alex C Speciale

**Affiliations:** Institute for Preventive Medicine and Public Health, Hanoi Medical University, 1 Ton That Tung, Kim Lien, Dong Da, Ha Noi 116001, Vietnam

**Keywords:** depression, stress, psychological distress, evolutionary mismatch, Vietnam, evolutionary medicine

## Abstract

**Background and objectives:**

Psychological distress is one of the greatest health threats facing humanity and has been hypothesized to represent an evolutionary mismatch. This hypothesis can be tested in semi-traditional societies that are undergoing transitions to modern lifestyles. This study used an evolutionary medicine framework to examine the predictors of psychological distress symptomology in a semi-modern ethnic minority village in rural Vietnam that is transitioning into a developing economy.

**Methodology:**

A cross-sectional survey was conducted in Chieng Sai Village among White Thai ethnic minority adults aged 18–75. The DASS-21 scale was used to measure the prevalence of psychological distress symptoms (depression and stress), and a closed format questionnaire was used to collect data on independent variables within an evolutionary mismatch framework. Binary logistic regression analyses were used to determine associated factors of psychological distress symptomology.

**Results:**

The prevalence of psychological distress symptoms was 22% (depression = 16.9%, stress = 16.3%). Common features of modernity, such as low levels of exercise, boredom, and low income, showed positive associations with psychological distress, while lifestyle features that were more similar to those expected in the evolutionary past and that fulfill evolutionary adaptations, such as getting enough sleep, adequate physical exertion, and access to resources (earning a sufficient income), showed negative associations with psychological distress.

**Conclusions and implications:**

This study suggests that modern lifestyles might have generated evolutionary mismatches that are negatively impacting mental health in Chieng Sai Village. Further investigations on mental health in rural Vietnam are warrented. Future research should focus on determining the causal relationship between psychological distress and evolutionary mismatches. Evolutionary medicine approaches to understanding and preventing psychological distress are potential forces of insight to be considered in public health and educational policy.

**Lay summary:**

Approximately 22% of White Thai ethnic minority adults in the village of Cheing Sai reported psychological distress symptoms. I found that lifestyle factors prevalent in modern society had positive associations with psychological distress symptomology, while lifestyle factors that mimic aspects of the human evolutionary past, such as adequate physical exertion, had negative associations with psychological distress symptoms.

## BACKGROUND AND OBJECTIVES

### Psychological distress as an evolutionary mechanism

Depression disorders, depression-like moods, chronic stress and acute stress are common forms of psychological distress. Depression is characterized by anhedonia, inactivity and suicidal ideation [1], whereas psychological stress is characterized by hyperactivity of the hypothalamic-pituitary-adrenal axis and the secretion of cortisol in a pulsatile pattern, in response to a real or perceived threat to homeostasis [2]. Non-communicable diseases often related to stress contribute to more than 68% of deaths worldwide and ∼75% of deaths in low- and middle-income countries. The Harvard TH Chan School of Public Health has predicted that 47 trillion dollars will be spent on stress-related non-communicable diseases in the next 20 years [3]. Depression is one of the most burdensome disorders world-wide and is the leading cause of disability for people aged 15–44 in the USA. As of 2018, an estimated 350 000 000 people world-wide were living with debilitating depression [4]. These conditions are particularly prevalent in developed civilizations [5], among graduate students [4], and especially among ethnic minority cultures that have been uprooted by development in such a way that indigenous cultures did not transition harmoniously into developed society [6].

The high prevalence of psychological distress in modern society may be the result of an evolutionary mismatch [7]. An evolutionary mismatch describes the negative consequences of disequilibrium that an organism experiences when traits that evolved to be adaptive in a previous environment become maladaptive in a new environment [6], or when ‘alleles that were previously favored are no longer favored in a new environment’ [8]. In order for an evolutionary mismatch to occur, the outcome in question does not need to be unique to the new environment; depression in modern society can still be caused by an evolutionary mismatch even if ancestral humans experienced depression to some degree.

Most of human existence has been spent in hunter-gatherer societies, thus humans are genetically adapted mostly for hunter-gatherer cultures and lifestyles [6]. The human genome has not changed much in 200 000 years, but due to the rapid transition from traditional society to modern society, our lifestyles have changed radically [6]. Psychological adaptations that were adaptive in ancestral environments can cause disease in modern environments [5, 6, 9]. Not surprisingly, as mental health issues are increasing in modern society, they are rarely found in hunter-gatherer societies that closely resemble the environments in which humans spent most of their time evolving [7, 9]. Perhaps the most convincing data that support the hypothesis that evolutionary mismatch is a key factor in psychological distress in modern society, is that a vast number of the known risk factors for psychological distress, were not present in our evolutionary past [6]. One example of a proposed evolutionary mismatch is related to postpartum depression, where modern day mothers often lack the social support that was present in ancestral times and that was critical for infant survival [9, 10].

It is thought that depressive symptoms after birth experienced by mothers evolved to function as signals for help with infant care when support was insufficient [11]. Consequentially, responses of social support to maternal signals for help would then prevent what is referred to in modern society as postpartum depression. In modern societies, where socially isolating culture does not provide immediate support in response to maternal signals for help, maternal distress is dysfunctional [11]. Hunter-gatherer families usually live in kin groups where grandparents, aunts, uncles, siblings and friends help mothers and fathers with childcare [12]. Dissimilarly, it is not uncommon for western nuclear families to be reduced in size and to live far away from kin compared with hunter-gatherers. The reduction of the nuclear family in modern civilization might negatively affect the mother’s ability to meet the demands of motherhood [12].

Psychological processes [9] like sadness, depression-like emotions [13, 14], and the functioning of the stress response [15, 16] are considered to be evolutionary mechanisms that offer adaptive advantages to humans under the right circumstances. The behavioral shutdown hypothesis of non-clinical depression posits that individuals should exhibit depressive like behavior, such as inactivity, in circumstances when activity would decrease their evolutionary fitness [14, 17]. Emotions that are commonly characterized by depression can make someone find what is lost, restore emotional attachment, and may cause individuals to accept or change unattainable goals while potentially conserving energy [13, 14, 18]. The stress response is critical for survival [15] and leads individuals to fight or to escape in threatening situations [19]. Under circumstances where depression-like emotions [20] and stress are not prolonged, they can be adaptive. When these adaptive functions are induced for prolonged periods of time, they can manifest in forms of depression disorders [6, 17] and stress-related illnesses [6, 15, 16, 19].

### Risk factors associated with post-agricultural lifestyles

Modern lifestyles may over-induce adaptive functions and lead to disease. In modern society, it is increasingly common that individuals work long hours indoors, are disconnected from social support, live sedentary lifestyles and occupy roles in society that are perceived as not meaningful or unfulfilling. Income inequality also exists across many highly developed civilizations [21]. Not surprisingly, long work hours [22], social isolation [15], lack of exercise [23], low perceived social status [24] and income inequality [21] have been shown to be risk factors for psychological distress. Furthermore, biome depletion, modern sanitation, and removal of species from the human biome may mediate mental illnesses through early neuroinflammatory events [6].

Viewing clinical depression and stress-related illnesses through the lens of an evolutionary mismatch is of particular importance because it allows us to see the complete etiology of the immense number of negative health consequences that are associated with psychological distress and that might propagate because of such a mismatch. Prolonged exposure to stress is linked to a plethora of unhealthy behaviors and illnesses such as substance abuse [25], high blood pressure, diabetes, cancer, obesity, heart disease [26] and clinical depression [27]. When left untreated, depression can lead to substance abuse [28], suicide, and suicide ideation [29]. The wide-ranging health impacts of modernity induced psychological distress may potentially be one of the greatest obstacles ever faced by medicine [6].

### Gaps and unique opportunities in Vietnam for studying psychological distress

There is a gap in psychological distress research on areas where semi-modern societies are culturally preserved and integrated into a market economy. Vietnam has preserved many of its traditional cultures amid a rapidly developing modern economy. It is home to 54 different ethnic minorities, many of whom occupy rural highlands and lowlands. Each ethnic group maintains their own distinct language and cultural traditions. Some minorities have been more exposed to development and modern amenities, while others live without access to amenities such as electricity and running water.

Depression is the second most common form of mental illness in Vietnam [4]. Approximately 40 000 Vietnamese people lose their lives due to depression each year [4]. Research on depression and stress has been done mostly in the major cities in Vietnam and has yielded similar trends to other international developed cities that show non-communicable diseases are linked to modern developed lifestyles. For example, provinces in Vietnam with a higher proportion of urban populations have higher mean levels of BMI and lower proportions of active people [30]. Additionally, the estimated percentage of mental health problems among undergraduate aged students in urban areas ranges anywhere from 25% to 60% [4]. A study on prevalence of anxiety and depression among secondary school students in Can Tho City reported numbers of 22.8% and 41.1%, respectively [29], while two prior studies on adolescents in Hanoi (*n* = 2591 and 1000) and one study on University students in Ho Chi Minh City (*n* = 410), revealed prevalence levels of depression at 26.3%, 16.2% and 36%, respectively [29]. With 70% of the Vietnamese population occupying rural populations, psychological distress research in rural villages in Vietnam remains scarce [30]. Although the pooled prevalence on hypertension in Vietnam shows a higher prevalence in mixed urban/rural settings compared with separate rural settings, hypertension is merely one example of a stress-related illness and information on associated factors is not widely available [30].

For the reasons outlined above, unique study paradigms arise in ethnic minority villages that adapt non-conflictingly into modern economies and that exist as semi-traditional societies. Vietnam has extraordinary opportunities to research villages that may offer insight into evolutionary lifestyle factors that could protect against psychological distress, and modernization factors that could predispose psychological distress. Chieng Sai Village is one of those villages. Due to development, these opportunities may not be finite.

### Objectives and rationale

One objective of this study was to measure the prevalence of psychological distress symptomology among White Thai ethnic minority adults in Chieng Sai Village, using depression and stress symptomology as indicators. Another objective was to identify novel associated factors of depression and stress symptoms in Chieng Sai using an evolutionary medicine framework. From an evolutionary perspective, since Chieng Sai Village is a semi-traditional village that has retained many of its cultural traditions in accordance with making a seemingly harmonious transition into development by embracing cultural tourism opportunities, I hypothesized that there may be unique lifestyle variables among Chieng Sai residents that mimic lifestyles of the environment of evolutionary adaptedness, and that such variables may show negative associations with depression and stress symptomology. For example, I expected factors that reinforce social cohesion (attending traditional social gatherings, engaging in social activities, living in a commune setting) and that stimulate biological rejuvenation (sleep and exercise) to show negative associations. Contrastingly, I proposed that factors influenced by modern lifestyles that conflict with the evolutionary needs of humans (being over-worked, earning an income that doesn’t provide adequate access to resources, low perceived social status, boredom, eating a modern diet) might have show positive associations with depression and stress symptomology. Identification of such factors would be useful for public health prevention and intervention efforts for psychological distress-related illnesses in Vietnam and abroad.

## METHODOLOGY

### Study design

This analytical cross-sectional study was conducted from April to May 2019 in Chieng Sai Village, Mai Chau Town, Mai Chau District, Hoa Binh Province, Vietnam.

Chieng Sai Village is located in a northwestern, mountainous region of Vietnam and is home to the White Thai ethnic minority, who have occupied the region since the first century CE. The White Thai subsist on wet rice cultivation and raising animals for consumption. Division of labor is equal and the language of the White Thai ethnic minority is taught verbally. Sixty percent of the White Thai ethnic minority can speak, read, and write in Vietnamese language, which is taught in primary and secondary schools. Many White Thai Ethnic minority residents of Chieng Sai Village still occupy traditional elevated wood houses that protect from landslides, flooding and predators.

The Pu Luong Nature Reserve is 25 km from the town of Mai Chau where Chieng Sai Village is located. The preserved cultural traditions of Mai Chau, and the serene beauty of the surrounding nature reserve attract national and international tourists alike. New hotels, homestays, restaurants and modern appliances shops are beginning to emerge throughout various parts of the town, creating a unique blend of traditional and non-traditional societal features. For example, although Chieng Sai farmers earn wages in the local currency and may consume modern appliances, many community members help each other when they plant and harvest their privately owned crops. In comparison, Bac Lac, a neighboring White Thai Village with a smaller population that is more isolated from development than Chieng Sai, retains a higher degree of egalitarianism and distribution of resources. There, many crops are communally owned and shared by village members. Additionally, in the face of development, attending love markets (traditional social gatherings to meet potential mates) has become increasingly less common for Chieng Sai villagers, as compared with the Hmong ethnic minority groups that occupy more isolated villages surrounding Mai Chau. Situations like this where the population is rapidly modernizing create a large amount of variation in modernization and can increase the power of finding an association with an outcome of interest.

Chieng Sai Village was chosen due to being an ethnic minority village that has been peacefully exposed to modernity and that seems to have used the market economy to supplement cultural preservation through tourism amid rapid development. Thus, Chieng Sai Village was a good candidate to investigate if a harmonious transition of traditional culture into modernity may retain ancestral-like lifestyle traits that fulfill evolutionary needs and protect against psychological distress. Additionally, Chieng Sai was a good candidate to investigate new ways in which factors related to an evolutionary mismatch might be associated with psychological distress. The lack of data in rural areas in Vietnam and the fact that these opportunities to study such villages may not be finite were also contributing factors for choosing Chieng Sai as a study site.

The total population of Chieng Sai Village was 1220 at the time data was collected, and data was collected over a duration of 3 days. White Thai ethnic minority adults aged 18–75 were surveyed for this study. No exclusion criteria beyond age and living location were utilized for recruiting participants for this survey. All participants were from Chieng Sai village. Convenience sampling and door to door sampling were used to recruit participants. For the pilot study and on the first day of the main survey, the village leader of Chieng Sai village utilized a community megaphone system to inform village residents to meet at the village cultural house to take part in the survey. On the last day of the main survey, door to door surveying was conducted.

Each participant gave their signed consent to take part in the study and received a payment amount equivalent to ∼1 USD for completing the survey. The survey was approved by the Hanoi Medical University committee for Master’s thesis proposals, by the local authorities in Mai Chau District, and by the village leader of Chieng Sai Village. A local White Thai informant assisted with obtaining approval from local authorities to conduct the study. I began building rapport with my informant and stakeholders in Mai Chau almost a year in advance to conducting the study. This study was designed using an evolutionary medicine framework to measure associated factors of psychological distress symptoms.

### Variables and indicators

Dependent variables consisted of depression and stress symptoms. Depression and stress symptoms were used as indicators of psychological distress because these are two major forms of psychological distress. The Depression, Anxiety, and Stress Scale- 21 items (DASS-21) sub scale scores were used as indicators for depression and stress symptoms. The DASS-21 scores (Table 2) were categorized as ‘Normal’, ‘Mild’, ‘Moderate’, ‘Severe’ and ‘Extremely Severe’, as formulated by the DASS-21 manual. Scores that fell under ‘Normal’ and ‘Mild’ were classified as ‘no presence of stress symptoms’ and ‘no presence of depression symptoms’, while scores that fell under ‘Moderate’, ‘severe’ and ‘extremely severe’ were classified as ‘presence of depressive symptoms’ and ‘presence of stress symptoms’.

**Table 2.** Prevalence of psychological distress and distribution of participants based on the categorization of their DASS-21 subscale scores according to DASS-21 manual

	Depression	Stress
No psychological distress	147 (83.1%)	148 (83.6%)
Normal	129 (72.9%)	128 (72.3%)
Mild	18 (10.2%)	20 (11.3%)
Psychologically distressed	30 (16.9%)	29 (16.4%)
Moderate	21 (11.9%)	19 (10.7%)
Severe	4 (2.3%)	4 (2.3%)
Extremely severe	5 (2.8%)	6 (3.4%)

Independent variables were allocated into separate categories: demographic factors (Table 1), social support and mate choice factors (Supplementary Table S1), sleep, diet and exercise factors (Supplementary Table S2), alcohol and tobacco consumption factors (Supplementary Table S3) and miscellaneous factors (Supplementary Table S4). Supplementary Tables S1–4 are included as supporting materials. Closed format questionnaires and the MacArthur Scale of Subjective Social Status were used as indicators for associated factors of psychological distress.

**Table 1.** Demographic variables of White Thai adults in Chieng Sai Village, Mai Chau, Hoa Binh, 2019

Variables	Frequency (%; *n* = 177)
Sex	
Male	78 (44.1%)
Female	99 (55.9%)
Age, mean (SD)	43.13 (12.42)
Age (years)	
18–25	18 (10.2%)
26–35	37 (20.9%)
36–45	41 (23.2%)
46–55	50 (28.2%)
56–75	31 (17.5%)
Marital status	
Single	19 (10.7%)
Married	146 (82.5%)
Divorced/widowed	12 (6.8%)
Educational status	
Primary school	25 (14.1%)
Secondary school	53 (29.9%)
High school	65 (36%)
College/University	34 (19.2%)
Occupation	
Farmer/laborer	121 (68.4%)
Restaurant/hotel/shop worker/student	16 (9.0%)
Police/government/city	19 (10.7%)
Other	21 (11.9%)
Workplace location	
Town	92 (52.0 %)
Village	54 (30.5%)
Both	27 (15.3%)
Salary per month	
0–3 Million	96 (54.2%)
≥ 4 Million VND	81 (45.8%)
Language	
Bilingual (Vietnamese and Thai)	162 (91.5 %)
Tri-Lingual (more than two languages)	15 (8.5%)

Aside from demographic variables, the independent variables measured in this study were selected using an evolutionary perspective on non-human primate and human health. Hunter-gatherers evolved in environments where being part of a socially supportive close-knit community, engaging in outdoor physical activities often, taking part in meaningful cultural experiences and occupying existentially fulfilling roles in society offered important benefits for survival [6]. Anthropological research on extant hunter-gatherer societies, who’s lifestyles are thought to resemble the lifestyles of prehistoric humans, suggests that sleep patterns would have been stable [31], and daily work hours would have been relatively shorter [32] and less stressful [6] compared with modernized daily routines.

Homeostasis and good health among our ancestors were maintained by multifactorial interactions involving the strategies mentioned above, and due to rapid niche transitions and the preservation of these beneficial strategies by natural selection, good health today still generally depends on the proper stimulation of the mentioned multifactorial interactions. Indeed, adequate social support [33], engaging in exercise [23], getting enough sleep [34] and not being overworked [35] are well established protective factors against psychological distress. On the other hand, features common in modern lifestyles, such as long working hours [22], social isolation [15], lack of exercise [23] and income inequality have been shown to be risk factors for depression and stress. Therefore, independent variables that measure these concepts and that are related to both ancestral and modern lifestyles were selected. For example, the variables that reflect ancestral states are included in the ‘social support and mate choice factors’, and ‘sleep, diet and exercise factors’ tables, while variables that reflect modern states are included in the ‘alcohol and tobacco factors’ table. Within the ‘miscellaneous factors’ table, ‘number of close friends’ and ‘social gatherings’ reflect ancestral states, and ‘subjective social status’, ‘hours of work per week’, ‘bored often’ and ‘most frequented location’ reflect modern lifestyles. Subjective social status was chosen as a variable related to modernity because modern societal structures are stratified by class, while ancestral societal structures are thought to have been egalitarian. Furthermore, prior studies have indicated that low perceived socioeconomic status is an indicator of adverse psychological distress outcomes [24]. Boredom was chosen as an independent variable related to modernity because modern working schedules can isolate people from exciting group interactions for many hours at a time. All independent variables were collected categorically except for ‘age’.

### Data collection tools and techniques

This study used a closed questionnaire format to measure demographic data and associated factors. The DASS-21 scale was used to measure depression and stress symptoms. The DASS-21 was chosen because it has previously been used in Vietnam to test validity among women in a rural setting. Overall, a high internal consistency was found for all subscales (depression, stress and anxiety) and the overall score [36]. Exploratory factor analyses and testing this scale against gold standard clinical interviews has indicated that the DASS-21 is a sensitive tool for screening for mental disorders among women with young children in rural northern Vietnam [36]. Given that the aim of this study was to estimate the prevalence of depression and stress symptoms, rather than to make clinical diagnoses, the DASS-21 screening instrument was an appropriate tool for this study.

A pilot study was carried out to check participant comprehension and face validity of the survey. Upon conducting the pilot study, no interpretational obstacles were found with the survey. During both the pilot study and main survey, three Vietnamese research assistants fluent in English and Vietnamese were present to check participant perception and to assist participants during the survey in the case that clarification on any survey item was needed. All three assistants were trained researchers with prior experience conducting surveys and work within the Department of Environmental Health at Hanoi Medical University. All scales and questionnaires were given in Vietnamese. Prior to conducting the pilot study and main survey, the questionnaires and scales were translated into Vietnamese by a Vietnamese college graduate who is fluent in English and Vietnamese. I was present during the translation to communicate with the translator and check that the translated version matched the concepts that were composed in English. Internal consistency was then checked by the Deputy Head of the Department of Environmental Health who is also fluent in Vietnamese and English, and has experience conducting depression research in Vietnam.

### Sample size

Village demographic data were obtained from the office of statistics and demographics in Mai Chau. The target population from Chieng Sai was 641. Prevalence levels for depression and stress symptoms obtained from the pilot study conducted prior to the main survey were found to be 19% and 18%, respectively. These proportions were used to calculate the sample size needed for the main survey. With a margin of error of 5%, a confidence level of 95% and a population size of 641, the needed sample sizes for depression and stress were 173 and 168, respectively.

### Statistical analysis

All data for this project were analyzed using IBM SPSS Statistics Subscription 1.0.0.1213. The calculation process described in the DASS-21 instructions was followed when scoring participant results. Depression and Stress both had seven questions each ranging from 0 to 3, with 0 being absent of symptoms, and 3 being a strong presence of symptoms. Scores were added and multiplied by 2 for each category. For depression, a score of 0–4 was considered normal, 5–6 = mild, 7–10= moderate, 11–13 = severe and 14 or more = extremely severe. For Stress, 0–7 = normal, 8–9 = mild, 10–12 = moderate, 13–16 = severe and 17 or more = extremely severe.

Binary logistic regression analyses were conducted to determine associated factors of depression and stress symptoms. Depression and stress were dichotomized according to the DASS-21 scale scoring and previous studies [37–39]. The outcome variables were dichotomized because the study was mainly concerned with how associations were related to the presence or absence of symptoms, rather than the severity of symptoms. The purposeful selection method [39] was used to help select independent variables to include in the model. First, a univariate analysis of each variable was conducted to check for significance (≤0.25). All significant variables were then added to the multivariate analyses. Next, variables were removed from the model if they were not found to be significant or confounders. This was done by evaluating any changes to parameters that remained in the model after variables were removed. After this, variables that have been well established in literature as risk factors for depression and stress, such as age and gender, were added to the model one by one to further check for confounding and significance. Non-confounders and non-significant variables were then removed, resulting in the final model. Questionnaires that contained missing data were not included in the analyses.

The prevalence of psychological distress, depression related symptoms and stress-related symptoms are displayed along with the distribution of participants according to the categorization of the DASS-21 subscale scores (Table 2). The results of the logistic regression analyses are reported as adjusted odds ratios (AORs), for which corresponding confidence intervals are attached. An α≤ 0.05 was considered as statistically significant for all statistics in the final analyses.

## RESULTS

### Participant characteristics

Overall, 187 participants completed the survey, however due to missing data and age disqualifications, the final sample put through analysis was 177 with 78 males (44.1%) and 99 females (55.9%). Adults aged 18–75 (*M* = 43.13, SD= 12.42) participated in the survey (Table 1).

### Prevalence of psychological distress, depression, and stress symptoms

Psychological distress was measured by depression and stress symptomology. The overall prevalence of psychological distress was 22%. The prevalence of depression symptoms was 16.9% (30 people). Approximately 72% of participants had normal symptoms of depression, 10.2% had mild symptoms, 11.9% had moderate symptoms, 2.3% had severe symptoms and 2.8% had extremely severe symptoms (Table 2).

The prevalence of stress symptoms was determined to be 16.3% (29 people). Similarly to depression symptoms, 72.3% of the sample had normal symptoms, 11.3% had mild symptoms, 10.7% had moderate symptoms, 2.3% had severe symptoms and 3.4% had extremely severe symptoms. Out of all the subtypes of psychological distress, ‘moderate’ was the most prevalent. Of the two subtypes for no psychological distress, ‘normal’ was most prevalent.

### Associated factors of depression symptoms

For depression, the omnibus Tests of Model Coefficients indicated the model was significant, χ^2^ (11, *n* = 177) =44.432, *P *<* *0.001, with between 22% and 37% percent of the variance in depressive symptoms being explained by the model (Nagelkerke $R^{2}$= 0.372). The Hosmer and Lemeshow Test was not significant, further supporting the model, χ^2^ (8, *n* = 177)= 10.128, *P *<* *0.256. Percentage Accuracy in Classification (PAC) = 87.6 (Table 3).

**Table 3.** Factors associated with depression symptoms among adults in Chieng Sai Village, Hoa Binh 2019

Depression: (χ^2^ (11, *n* = 177) =44.432, *P *<* *0.001, Nagelkerke $R^{2}$= 0.372)				
	*P* value	AOR	95% CI for AOR	
			Lower	Upper
Alcohol use				
Does not consume alcohol (ref.)				
Consumes alcohol	0.028	0.290	0.096	0.874
Hours of work per week				
0–25 h ref.)	0.065			
26–40 h	0.235	7.53	0.268	211
41–56 h	0.052	25.7	0.978	676
57 or more	0.042	34.8	1.13	1071
Monthly income				
0–3 million (ref.)				
≥ 4 million VND	0.010	0.225	0.073	0.697
Hours of mild exercise per week				
<2.5 h (ref.)	0.002			
2.5–3.5 h	0.009	0.033	0.003	0.423
4–5 h	0.007	0.066	0.009	0.471
>5 h	0.002	0.157	0.048	0.511
Hours of sleep per night				
0–6 h (ref.)	0.005			
6.5–8 h	0.035	0.342	0.125	0.929
8.5–10 h	0.030	10.7	1.26	91.2
Perceived boredom				
Bored often (ref.)				
Not bored often	0.043	2.73	1.03	7.23

Abbreviations: AOR, adjusted odds ratio; CI, confidence interval; ref., reference group; χ^2^, Chi-square.

Alcohol use, hours of work per week, monthly income, hours of mild exercise per week, hours of sleep per night and perceived boredom were all shown to be factors associated with depressive symptoms. Interestingly, participants who consumed alcohol had lower odds (AOR= 0.290; 95% CI 0.096, 0.874) of experiencing depressive symptoms than participants who did not consume alcoholic beverages. In addition, as work hours increased, so did the odds of depressive symptoms. Participants who worked 57 h or more per week had higher estimated odds (AOR= 34.8; 95% CI 1.13, 1.071) of depressive symptoms than participants who worked 0–25 h/week.

Increased odds of depression symptoms were also shown for participants who worked between 26 and 40 h (AOR= 7.53; 95% CI 0.268, 0.211), and between 41 and 56 h/week (AOR= 25.7; 95% CI 0.978, 0.676); however, the confidence intervals crossed through 1. Participants earning an income of 200 USD or more per month also had lower odds of reporting depressive symptoms (AOR= .225; 95% CI 0.073, 0.697) than participants who earned an income of 0–150 dollars per month.

As level of mild exercise per week increased, the odds of depressive symptoms decreased. Respondents who exercised 2½–3½ h/week had lower odds (AOR= 0.033; 95% CI 0.003, 0.423) of having depression symptoms than those who exercised <2½ per week. Participants who exercised 4–5 h/week had lower odds (AOR= 0.066; 95% CI 0.009, 0.471) of exhibiting depressive symptoms compared with villagers who got <2½ h of mild exercise per week, while participants who got >5 h of mild exercise per week also had lower odds (AOR= 0.157; 95% CI 0.048, 0.511) of showing depression symptoms compared with the reference group.

Participants who slept 6½–8 h/night had lower odds (AOR= 0.342; 95% CI 0.125, 0.929) of experiencing depressive symptoms than those who slept from 0 to 5 h/night. Interestingly, participants sleeping from 8½ to 10 h/night had higher odds (AOR= 10.7; 95% CI 1.26, 91.2) of exhibiting depressive symptoms compared with participants who reported sleeping from 0 to 6 h/night. Perceived boredom was also associated with depressive symptoms. Participants who reported being bored often had higher odds (AOR= 2.73; 95% CI 1.03, 7.23) than those who were not bored often.

### Associated factors for stress

Similarly, for stress, the omnibus tests of model coefficients indicated the model was significant, χ^2^ (12, *n* = 177) =46.688, *P *<* *0.001, with between 23% and 39% of the variance in stress symptoms being explained by the model (Nagelkerke $R^{2}$= 0.393). The Hosmer and Lemeshow test was not significant, further supporting the model, χ^2^ (8, *n* = 177) =7.328, *P *=* *0.502. PAC = 86.4 (Table 4).

**Table 4.** Factors associated with stress symptoms among adults in Chieng Sai Village, Hoa Binh 2019

Stress: (χ2 (12, N=177) =46.688, P<0.001, Nagelkerke $R^{2}$= 0.393)				
	*P* value	AOR	95% CI for AOR	
			Lower	Upper
Alcohol use				
Does not consume alcohol (ref.)				
Consumes alcohol	0.004	0.154	0.043	0.549
Hours of mild exercise per week				
<2.5 h (ref.)	0.010			
2.5–3.5 h	0.101	0.251	0.048	1.31
4–5 h	0.007	0.041	0.004	0.426
>5 h	0.007	0.176	0.050	0.624
Perceived boredom				
Bored often (ref.)				
Not bored often	0.001	5.52	1.95	15.6
Subjective social status				
Low (0–3) (ref.)				
Average/high (4–9)	0.060	0.359	0.124	1.04

Abbreviations: AOR, adjusted odds ratio; CI, confidence interval; ref., reference group; χ^2^, Chi-square.

Alcohol use, hours of mild exercise per week and perceived boredom were found to have significant associations with stress symptoms. Similarly, with depression symptoms, participants who consumed alcoholic beverages had lower odds (AOR= 0.154; 95% CI 0.043, 0.549) of experiencing stress symptoms compared with those who did not consume alcohol. Respondents who got 4–5 h of mild exercise per week had lower odds (AOR= 0.041; 95% CI 0.004, 0.426) of displaying stress symptoms in the last week than those who got <2½ of mild exercise per week, and participants who reported getting >5 h of mild exercise per week had lower odds (AOR= 0.176; 95% CI 0.050, 0.624) of experiencing stress symptoms.

Villagers who reported being bored often had higher odds (AOR= 5.52; 95% CI 1.95, 15.6) of experiencing stress symptoms compared with those who were not bored often. Last, individuals who reported a subjective social status between four and nine on the measurement ladder had lower odds (AOR= 0.359; 95% CI 0.124, 1.04) of displaying stress symptoms than individuals who had a subjective social status score from 0 to 3. However, the CI crossed through one.

## DISCUSSION

As demonstrated in prior research on psychological distress from industrialized societies [7], the factors that showed positive associations with psychological distress symptoms in Chieng Sai Village seem to be factors related to modern development, which is not surprising. For example, working long hours, low levels of exercise and not getting enough sleep were positively associated with psychological distress symptoms in the sample. These are factors that disrupt the evolutionary adaptations of humans [5, 6]. Getting more than 8.5 h of sleep showed a positive association with depression, which may be explained by depression leading to hypersomnia [40].

Participants who reported higher levels of exercise had lower odds of displaying psychological distress symptoms, compared with individuals who reported getting low levels of exercise. Likewise, a systematic review of 30 cohort studies concluded that physical activity conferred protective advantages against depression in 25 out 30 reviewed studies [41]. The hormones released by stimulating physical exercise are known to combat against depression, and are likely incentives maintained by natural selection for achieving goals or obtaining rewards that required engaging in physical activity; engaging in activities such as migrating, hunting, or fighting were important for survival in the evolutionary history of human beings. In Chieng Sai, it is not clear to what extent the benefits associated with physical activity arose from free time physical activity vs. labor related physical activity. Future studies should focus on distinguishing between these sources of physical activity.

It is also important to point out that behaviors unique to modern lifestyles can stimulate evolutionary physiological pathways and produce health benefits even if ancestral humans did not partake in those specific activities. For example, modern day humans can gain the same health benefits by going on a hike that ancestral humans may have gained through foraging or going on a hunt; both activities are forms of physical exertion that stimulate metabolic pathways and are beneficial for health [42]. Alcohol consumption and the health benefits of socializing may be mediated in a similar manner.

Alcohol consumption in Chieng Sai was found to have a negative association with psychological distress among participants who displayed both depression and stress symptoms. Similar findings from a 7-year cohort study (*n* = 5505) support this connection [43]. Other studies suggest it is possible that socializing is a confounding variable in this situation, since individuals who consume alcohol may do so as a function of socializing and reducing stress [44]. This could be an example of a modern behavior producing health benefits by activating evolutionary physiological pathways related to bonding. However, when socializing was controlled for in the sample, there remained an independent negative association between depression and psychological distress. Another explanation for the negative association between psychological distress and alcohol consumption might be the U-dose response to alcohol [45]. Further research is needed to explain this connection.

Interestingly, age, gender, level of social support and level of education showed no associations to psychological distress in Chieng Sai Village. This is particularly interesting information, because in much of the published literature on psychological distress in modern societies, disparate measures of psychological distress exist among these variables. For example, a recent cross-sectional study among students aged 13–17 from four different geographical locations in Vietnam found that female students and older students had a higher risk of experiencing depressive symptoms than younger male students [46]. Another cross-sectional study among the Danish general population also found that females were more likely to have depression [47]. For gender, one explanation may be that gender roles in Chieng Sai are more equally distributed in society, with men and women both occupying prominent roles. Age may not be an associated factor of psychological distress in Chieng Sai Village because elders may have more meaningful roles in the family than elderly individuals do in modern society. Numerous studies from modern societies indicate that social support is critical for maintaining psychological and physiological health [48]. Social support may not have been an identifiable associated factor due to the low number of respondents who reported not having social support. Future qualitative and quantitative research may help reveal how these variables are mediated within Chieng Sai and such an understanding could lend insight into how to reduce disparity of psychological distress in developed nations where age and gender are risk factors of psychological distress.

Level of education in Chieng Sai village showed no significant association with psychological distress. Prior studies from abroad have yielded various results across different populations. For example, in a cohort of 33 774 individuals, a low level of education was significantly related to depression and anxiety [49]. Because the human organism has evolved to rely heavily on the transfer of niche specific information in order to avoid health complications, it would make sense that a high level of education (a system designed to transfer knowledge) would protect individuals from psychological distress by providing individuals with the theoretical and practical tools that are necessary to prevent experiencing complications from such conditions. At first glance it is hard to ascertain why a high level of education could have a positive, or even a neutral relationship to psychological distress. In Chieng Sai, and in societies that show education level has no association to psychological distress, education may not be an associated factor because educational curriculum may not be relevant to successfully thriving in certain environments. For example, because a large percentage of Chieng Sai residents are farmers who are able to make a decent living and don’t rely on learning farming in school to be a successful farmer, level of education may have no relevance to one’s ability to successfully subsist in Chieng Sai. Inversely, complex societies that require a lot of education to achieve successful health outcomes may be burdened by psychological distress if the school curriculums are not adequately designed to prepare individuals for success in their niches.

### Limitations

This study had some limitations that must be considered. First, care should be taken when drawing causal conclusions from cross-sectional data. This study does not draw any definitive causal conclusions regarding psychological distress in Chieng Sai. A second limitation is that all independent variables were self-reported. Asking participants to self-report desirable/undesirable behaviors may generate biased responses. Additionally, this study only measured psychological distress through two conceptual measures: depression and stress. It is possible that the prevalence of psychological distress in Chieng Sai is higher if other forms of psychological distress are present. Next, this study had no role in clinical diagnoses of participants. The DASS-21 is a screening instrument, meaning that the prevalence levels described in this study are estimates of the proportion of people who may be negatively affected by various levels of psychological distress in Chieng Sai Village. Another limitation was the sampling method, which could have produced an under or over estimation of psychological distress in the population.

Another consideration is that Chieng Sai village is one of many white Thai ethnic minority communities in Vietnam; thus, the results from this research can only speak to Chieng Sai Village alone. Psychological distress may be more or less prevalent in other White Thai villages due to environmental or cultural factors unique to other regions, and associated factors may be different too. Last, some of the reported associated factors and questionnaire items yielded a low number of responses, which produced wide confidence intervals, making some of the estimated AORs difficult to ascertain. Regardless of the limitations, the prevalence levels and associated factors found in this study are significant findings with important public health implications. This is also the first study to use the DASS-21 to investigate psychological distress among White Thai Ethnic Minority male and female adults from a mixed urban/rural population.

## CONCLUSION AND IMPLICATIONS

Protective factors for psychological distress in Chieng Sai Village are linked to lifestyle characteristics that mimic the environment of evolutionary adaptedness (hours of sleep per night) or that stimulate evolutionary adaptive pathways, even though the behaviors themselves might be unique to modernity (income and exercise). Despite these protective factors, and given the effectiveness of the DASS-21 for screening for psychological distress, the results from this study may suggest that the White Thai ethnic minority residents of Chieng Sai village have been negatively affected by common features of modernity and an evolutionary mismatch. In Vietnam, and abroad, other ethnic minority villages who integrate non-forcefully into development may also be negatively impacted in a similar way by evolutionary mismatches. Future research is needed on such villages and Vietnam presents unique research opportunities. Treatment and prevention strategies in villages like Chieng Sai should be quickly mobilized while population size is small and disease burden is manageable. Long-term prevention strategies for psychological distress might be achieved by the construction of niches that harmonize with the evolutionary adaptations of humans and might benefit by integrating niche specific information into educational curriculum. For proximate treatment strategies for psychological distress, evolutionary medicine approaches might be effective. Treatment strategies should consider how one’s niche or lifestyle can be altered to eliminate prolonged psychological distress through exposures to protective factors that either directly mimic the environment of evolutionary adaptedness or use modern behaviors to activate the evolutionary physiological pathways upon which good health depend, such as getting enough sleep, physical exertion, social bonding, finding hobbies to reduce boredom, securing access to resources and not being overworked. Evolutionary medicine approaches to treating psychological distress conditions may be a cost-effective and safer alternative to treatment modalities such as treatment with Selective Serotonin Reuptake Inhibitors.

## Supplementary data

Supplementary data is available at *EMPH* online.

## Supplementary Material

eoab014_Supplementary_Data
