# Supplementary material for: Prevalence and factors related to psychological distress among ethnic minority adults in a semi-modern village in rural Vietnam: an evolutionary mismatch framework
Source: Evol Med Public Health. 2021 Apr 30;9(1):194–205. doi: 10.1093/emph/eoab014 (PMC8494111; doi:10.1093/emph/eoab014)
Supplement: eoab014_Supplementary_Data [file eoab014_supplementary_data.pdf]

Table S1. Social support and mate choice factors among adults in Chieng Sai Village, Hoa Binh 2019

Variables		Frequency (%) (N=177)
**Familial Support**		
	Yes	160 (90.4%)
	No	17 (9.6%)
**Support from friends**		
	Yes	161 (91.0%)
	No	16 (9.0%)
**Spousal Support**		
	yes	151 (85.3%)
	No	25 (14.1%)
**Perceived Access to Mates**		
	Yes	26 (14.7%)
	No	9 (5.1%)
**Desire to relocate**		
	Yes	10 (5.6%)
	No	167 (94.4%)

Table S2. Exercise, Diet, and Sleep

Variables		Frequency (%) (N=177)
**Hours of mild exercise per week**		
	Less than 2.5 Hours	62 (35.0%)

2.5-3.5 Hours	29 (16.4%)
4-5 Hours	19 (10.7%)
More than 5 hours	67 (37.9%)
**Hours of Intense exercise per week**	
Less than 30 Minutes	62 (35.0%)
1-2 Hours	42 (23.7 %)
3-4 Hours	20 (11.3%)
5-6 Hours	20 (11.3%)
More than 6 Hours	33 (18.6%)
**Diet**	
Natural grown food	154 (87.0%)
Processed foods	12 (6.8%)
Both	11 (6.2%)
**Hours of Sleep Per Night**	
0-6 Hours	74 (41%)
6.5-8 Hours	95 (53%)
8.5-10 Hours	8 (4.5%)

Table S3. Tobacco and Alcohol Consumption

**Variables**	**Frequency (%) (N=177)**
**Alcohol Consumption**	
Yes	65 (36.7%)
No	112 (63.3%)
**Cigarette Smoking**	
Yes	32 (18.1%)

No	145 (81.9%)
**Water Pipe Use**	
Yes	16 (9.0%)
No	161 (91.0%)

Table S4. Miscellaneous Variables

Variables	Frequency (%) (N=177)
**Perceived social status**	
Low (0-3)	40 (22.6%)
Average/High (4-9)	137 (77.4%)
**Hours of work per week**	
0-25 Hours	10 (5.6%)
26-40 Hours	49 (27.7%)
41-56 Hours	93 (52.5%)
57 or more	25 (14.1%)
**Most Frequented Location**	
Town	105 (59%)
Village	67 37.9%)
**Number of close friends**	
0-5	105 (59.3%)
6-10	42 (23.7%)
11 or more	30 (16.9%)
**Social gatherings**	
Yes	129 (72.9%)
No	48 (27.1%)

**Bored Often**		
	Yes	71 (40.1%)
	No	106 (59.9%)
